# Inversely polarized thermo-electrochemical power generation via the reaction of an organic redox couple on a TiO_2_/Ti mesh electrode

**DOI:** 10.1038/s41598-021-93269-7

**Published:** 2021-07-06

**Authors:** Hiroto Eguchi, Takashi Kobayashi, Teppei Yamada, David S. Rivera Rocabado, Takayoshi Ishimoto, Miho Yamauchi

**Affiliations:** 1 Department of Chemistry, Graduate School of Science, Kyushu University, Motooka 744, Nishi-ku, Fukuoka, 819-0395 Japan; 2 Division of Applied Chemistry, Graduate School of Engineering, Kyushu University, Motooka 744, Nishi-ku, Fukuoka, 819-0395 Japan; 3 Center for Molecular Systems, Kyushu University, Motooka 744, Nishi-ku, Fukuoka, 819-0395 Japan; 4 Graduate School of Nanobioscience, Yokohama City University, Seto 22-2, Kanazawa-ku, Yokohama, 236-0027 Japan; 5 Smart Innovation Program, Graduate School of Advanced Science and Engineering, Hiroshima University, Kagamiyama 1-4-1, Higashi-Hiroshima, Hiroshima, 739-8527 Japan; 6 International Institute for Carbon-Neutral Energy Research (WPI-I2CNER), Kyushu University, Motooka 744, Nishi-ku, Fukuoka, 819-0395 Japan; 7 Advanced Institute for Materials Research (WPI-AIMR), Tohoku University, 2-1-1 Katahira, Aoba-ku, Sendai, 980-8577 Japan

**Keywords:** Energy science and technology, Thermoelectric devices and materials

## Abstract

We demonstrate thermo-electrochemical (TEC) conversion using a biocompatible redox couple of lactic acid and pyruvic acid on earth-abundant TiO_2_. The TEC cell exhibited a positive Seebeck coefficient of 1.40 mV K^−1^. DFT calculations figured out that the adsorption of intermediate species and protons on TiO_2_ controls both the redox reaction and current polarity.

## Introduction

The thermoelectric conversion is a powerful tool to upgrade widely distributed low-grade heat energies, which are characterized by temperatures lower than 200 °C, such as waste heat, geothermal source, and solar heat, into electricity^1^. In the past, solid-state materials have been applied for the thermoelectric conversion, e.g., inorganic materials^2^, polymer matters^3^, and organic–inorganic hybrid materials^4^. Recently, thermo-electrochemical (TEC) cells, which produce electric power using the temperature differences of an electrochemical cell containing a redox couple, have attracted considerable attention due to their high Seebeck coefficient (*S*_e_) in the relatively low-temperature region. Electric potential is generated via the redox reaction described as:

1 $$\mathrm{A}+\mathrm{n}{\mathrm{e}}^{-}\,\,\to \mathrm{B}$$

with a *S*_e_ value expressed by^5,6^

2 $${S}_{\mathrm{e}}=\frac{\Delta\,\,V}{\Delta\,\,T}\,\,=\,\,\frac{{S}_{\mathrm{B}}-{S}_{\mathrm{A}}}{nF}$$

where *V* is the electric potential, *T* is temperature, *S*_A_ and *S*_B_ are the partial molar entropies of A and B, *n* is the number of electrons involved in the reaction, and *F* is the Faraday constant. High *S*_e_ values in the order of mV K^−1^ are achievable due to the large temperature dependence of equilibrium potentials for the reaction involving a multielectron redox reaction. Ferri/ferrocyanide (Fe(CN)_6_^3−^/Fe(CN)_6_^4−^), which has a *S*_e_ value of − 1.4 mV K^−1^ is one of the typical redox couples^7^. Moreover, various redox couples were investigated, e. g., iodide/triiodide(I^−^/I_3_^−^)^8^ and cobalt(II/III) tris(bipyridyl) (Co^2+/3+^ (bpy)_3_)^9^ redox couple. The reported *S*_e_ value are listed in Table S1. Due to the increasing demand for the thermoelectric conversion, TEC cells with inexpensive and abundant materials are required. Until now, organic redox couples such as quinone/hydroquinone (quinhydrone)^10^ and acetone/isopropanol^11^ have been applied to TEC applications and large negative *S*_e_ values in organic redox couples system were obtained, e.g. − 0.63 to − 9.9 mV K^−1^.^10,11^ However, the carcinogenicity of quinhydrone or air sensitivity of isopropanol are drawbacks of these systems.

**Figure 1:**
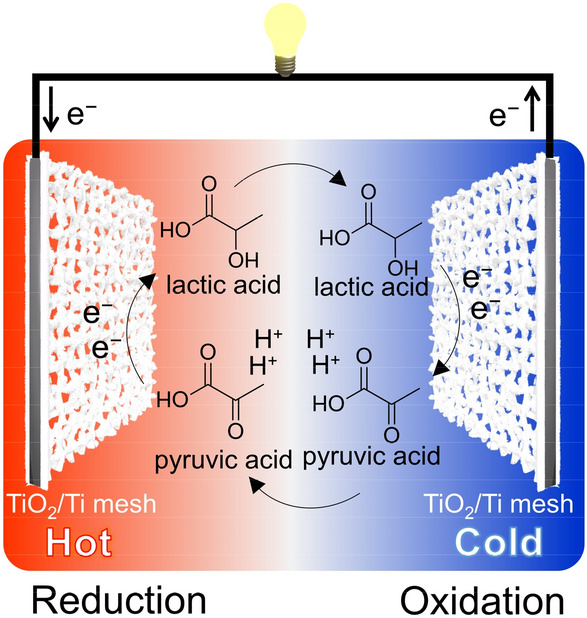
A scheme of thermo-electrochemical (TEC) cell using pyruvic acid and lactic acid as a redox couple and TiO_2_/Ti mesh electrodes. Oxidation (or reduction) reaction proceeds at the cold (or hot) side.

Considering the high efficiency of TEC cells in the low-temperature range, e.g., systemic temperatures, TEC conversion using body heat is a promising application^12^. In this regard, high biocompatible redox couples are needed, although few works for biocompatible couples have been performed. Herein, we focus on the TEC conversion using an organic redox couple composed of pyruvic acid and lactic acid. These compounds are ingredients in the metabolic system and intrinsically non-toxic. Recently, we have reported that α-keto acids are electrochemically reduced into corresponding α-hydroxyl acids on TiO_2_ electrocatalyst with high efficiency^13–19^. In this report, we demonstrate TEC conversion employing a biocompatible redox couple of pyruvic acid and lactic acid, with the support of TiO_2_ catalysts grown on a Ti mesh electrode (TiO_2_/Ti mesh electrode)^15–19^ as shown in Fig. 1. TEC cells were generally composed of expensive materials such as carbon nanotubes^20^ and platinum.^21^ Utilization of earth-abundant materials such as TiO_2_ will accelerate the application of TEC cells.

## Results and discussion

**Figure 2:**
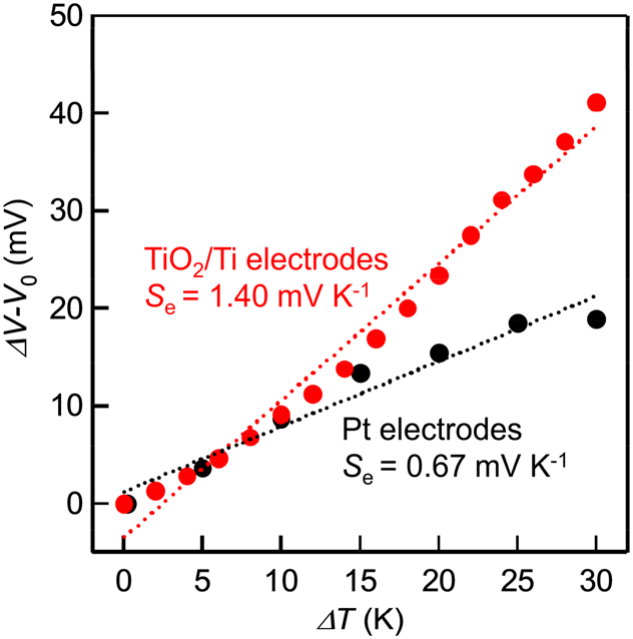
Difference in the open-circuit potential (*ΔV* − *V*_0_) and temperature difference (*ΔT*) of the TEC cell using TiO_2_/Ti mesh electrodes (red) and Pt wire electrodes (black). *V*_0_ is the initial open-circuit potential differences, as shown in Table S2. The initial concentration of pyruvic acid, lactic acid, and sodium sulfate were 20, 20 and 50 mM, respectively. Seebeck coefficients were calculated by the least-square fitting of a dotted line.

The TiO_2_/Ti mesh electrode was prepared according to our previous report^15^. The XRD pattern shown in Fig. S1 represents that anatase type TiO_2_ is contained in the electrode. The red plot in Fig. 2 and Fig. S2 show a difference in open-circuit potential (*ΔV* − *V*_0_) plotted to the temperature difference between two electrodes (*ΔT*) in the TEC cell, where *V*_0_ is the initial open-circuit potential difference at *ΔT* = 0. *ΔV* − *V*_0_ was calculated using the difference in the potential between two electrodes (Fig. S3). The larger *ΔT* resulted in the larger *ΔV*-*V*_0_, which ensures conversion from heat energy to electrical power. We estimated the *S*_e_ value by the least square analysis of *ΔV* − *V*_0_ varying with *ΔT* (dashed line in Fig. 2) to be 1.40 mV K^−1^, which is a positive *S*_e_ value. In contrast to the TEC conversion as shown in Fig. S4(a), the proportional relationship between *ΔV* − *V*_0_ and *ΔT* was not observed without pyruvic acid and lactic acid as indicated in Fig. S4(b). The deviation in the experiments without the organic acids reflects the instability of the redox potential with the very low concentration of the redox molecules. The black plot in Fig. 2 displays *ΔV* − *V*_0_ plotted to *ΔT* of a TEC cell consisting of platinum electrodes. We observed a proportional relationship between *ΔV* − *V*_0_ and *ΔT* and determined the *S*_e_ to be 0.67 mV K^−1^, which is less than one half of the *S*_e_ value for the cell using TiO_2_/Ti mesh electrode, indicating catalytic natures of TiO_2_ give positive effects on the TEC conversion.

Equation (2) represents that the electric potential change on electrodes resulting from the temperature difference is ascribed to entropy change in a common redox reaction. The redox reaction involving pyruvic acid and lactic acid is described as the following equation.

3 $$\mathrm{C}{\mathrm{H}}_{3}\mathrm{C}\mathrm{O}\mathrm{C}\mathrm{O}\mathrm{O}\mathrm{H}+2{\mathrm{H}}^{+}+2{\mathrm{e}}^{-}\,\,\rightleftharpoons\,\,\mathrm{C}{\mathrm{H}}_{3}\mathrm{C}\left(\mathrm{O}\mathrm{H}\right)\mathrm{H}\mathrm{C}\mathrm{O}\mathrm{O}\mathrm{H}$$

Equation (3) suggests that the entropy decreases along with the progress of the reduction of pyruvic acid. The standard Gibbs energies of formation for pyruvic acid and lactic acid are − 351.18 and − 314.49 kJ mol^−1^^22^, and standard enthalpies of formation for pyruvic acid and lactic acid are − 596.84 and − 687.88 kJ mol^−1^, respectively^22^. From these thermodynamic parameters, the *S*_e_ value for the TEC system employing the redox reaction between pyruvic acid and lactic acid was estimated to be − 2.20 mV K^−1^. This negative *S*_e_ leads to the progress of oxidation (or reduction) reaction at the hot (or cold) side, which is in contrast to the positive *S*_e_ value obtained in this system. Consequently, oxidation of lactic acid (or pyruvic acid reduction) proceeds at the cold (or hot) side in our TEC system. Previously, we recognized that the TEC cell with Pt electrodes resulted in the different *S*_e_ values from that with TiO_2_/Ti electrodes, which suggests that the reaction mechanism depends on the materials used for the electrodes.

**Figure 3:**
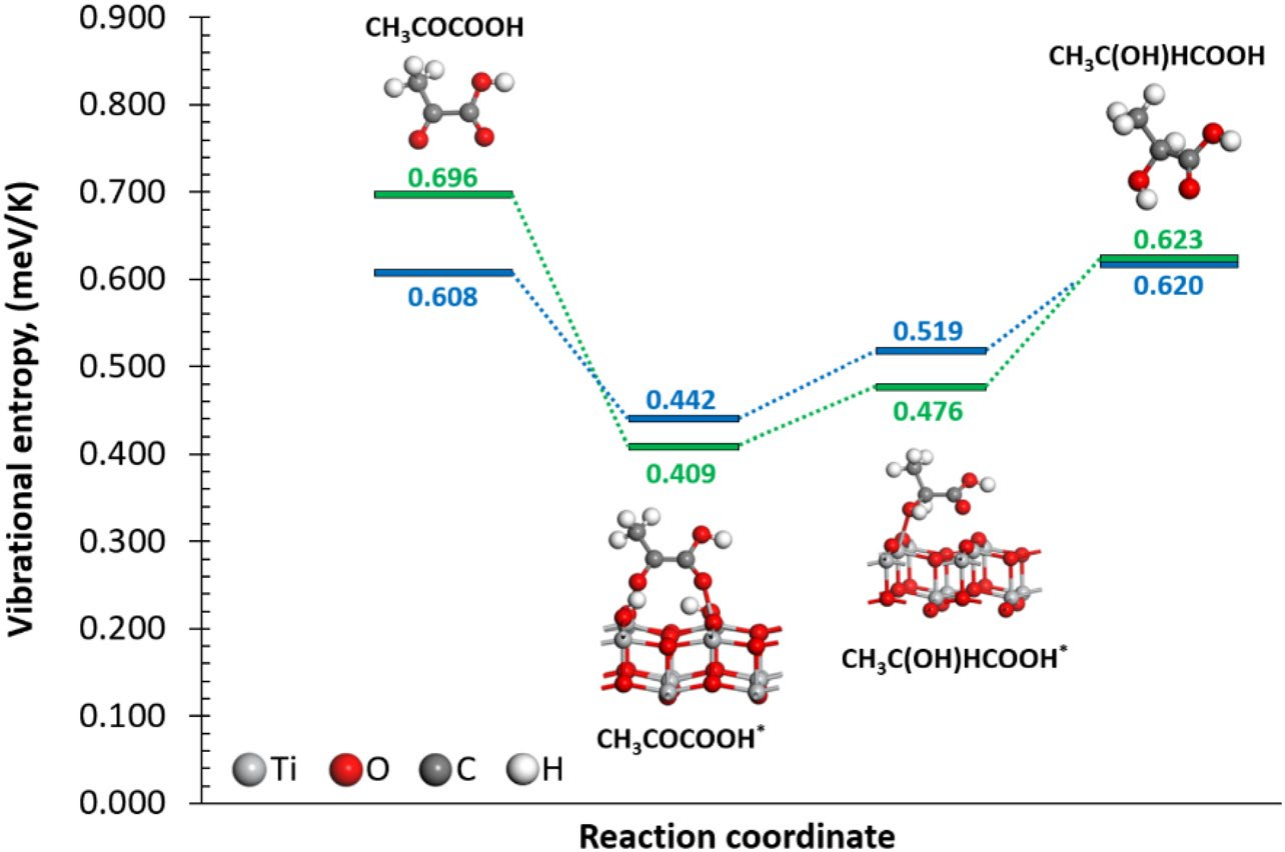
Vibrational entropies of the isolated and adsorbed pyruvic and lactic acid molecules. The green and blue paths correspond to the entropies without and with solvent effects, respectively.

To investigate the reaction mechanism that shows the positive *S*_e_ value in this TEC system, density functional theory (DFT) calculations with solvent effect were performed and the reaction energy change between lactic acid and pyruvic acid on the TiO_2_ surface was investigated. As a reaction field of pyruvic acid, the TiO_2_(001) surface (Fig. S5) with two adsorbed H atoms is assumed. The thermodynamically stable interaction of two H atoms with two of the TiO_2_(001) surface twofold coordinated O (O_2c_) atoms leads to the formation of two OHs, as shown in Fig. S6a. Then the pyruvic acid reduction reaction is studied on the TiO_2_(001) surface with the pre-adsorbed H atoms as an initial state. The most stable interaction of the pyruvic acid molecule on the protonated TiO_2_(001) surface is the molecule with each of its uncoordinatedly saturated O atoms interacting with two surface Ti atoms closest to the already formed OHs. The relative energy of the adsorption of pyruvic acid on the protonated TiO_2_ is − 0.816 eV. The reaction pathway of the pyruvic acid adsorption and its reduction to lactic acid by the sequential association of H atoms from the protonated TiO_2_(001) surface is shown in Fig. S7. The first H association leading to the formation of CH_3_COHCOOH is an exothermic process with relative energy of − 0.846 eV. The association of the remaining H atom on the surface to the CH_3_COHCOOH leads to lactic acid formation. Compared to the first association, the second association is an endothermic process; the relative energy corresponding to the lactic acid formation is − 0.180 eV. The energy changes during the reaction were larger when the solvent effect is not considered, as shown in Fig. S7. Figure 3 shows the vibrational entropies of the isolated and adsorbed substrate molecules with and without solvent effects. The calculated vibrational entropies of the isolated pyruvic (0.608 meV K^−1^) and lactic (0.620 meV K^−1^) acids are larger than the values of the adsorbed molecules, 0.442 meV K^−1^ for the pyruvic acid and 0.519 meV K^−1^ for the lactic acid. The entropy difference between isolated and adsorbed molecules is just a reflection of the conversion of free translational/rotational/vibrational degrees of freedom into bound motions^23^. Based on these theoretical results, the positive relative energy, 0.636 eV, the oxidation of lactic acid is more energetically feasible than the pyruvic acid reduction. On the other hand, the vibrational entropy difference, 0.077 meV K^−1^, between the adsorbed pyruvic and lactic acids was obviously positive, although isolated molecules are almost the same. Based on the vibrational entropy difference, the calculated *S*_e_ can be approximated to a value of 0.038 mV K^−1^. The positive *S*_e_ value was obtained from the DFT calculations as well as from the experimental results. This result indicates that the existence of TiO_2_ plays an essential part in showing a positive *S*_e_ sign. Additionally, one of the roles of the TiO_2_ surface is the H donor/acceptor during the oxidation/reduction reactions. However, the large difference between the experimental and calculated *S*_e_ values remains. Although we found the importance of the solvent effect during the reaction. A reason for the discrepancy between theoretical and experimental results is that the solvent is treated as a structureless continuum with certain dielectric and interfacial properties.^24^ In an explicit solvent model, the molecules can contribute over 90% of atoms in a simulated system, as a result, the number of interacting particles and the number of degrees of freedom of a system, and hence the entropy in the implicit models are significantly reduced.^25^ Moreover, leaving out the explicit description of the solvent comes at the cost of lacking hydrogen bonds with solvent, overstabilized salt bridges, and hydrogen bonds within the solute, etc^25,26^. Other sources of discrepancy can be due to the homogeneity of the slab model considered in this work compared to the heterogeneity of the nanoparticles as the reaction might occur at ridge, vertex, or different facets. In the same line, the surface roughness, such as defects, is unclear for TiO_2_ catalyst in our experiment. The presence of pre-adsorbed species, i.e., the coverage effect, also affects the change in entropy; for the carbolac-ethyl chloride system, at the start of the adsorption, there is an increase in entropy as the molecules are adsorbed in a disorderly array. In our calculation, we also found that when using TiO_2_ surface without H adsorption, the calculated *S*_e_ value between adsorbed pyruvic and lactic acids is smaller, 0.027 mV K^−1^. Then, as the surface becomes covered by a monolayer and the molecules are very orderly arranged, the entropy decreases. Finally, in the region of multilayer adsorption, there is a small entropy decrease that approaches zero. These three regions have been observed for the entropy changes in a variety of adsorption systems^27^.

**Figure 4:**
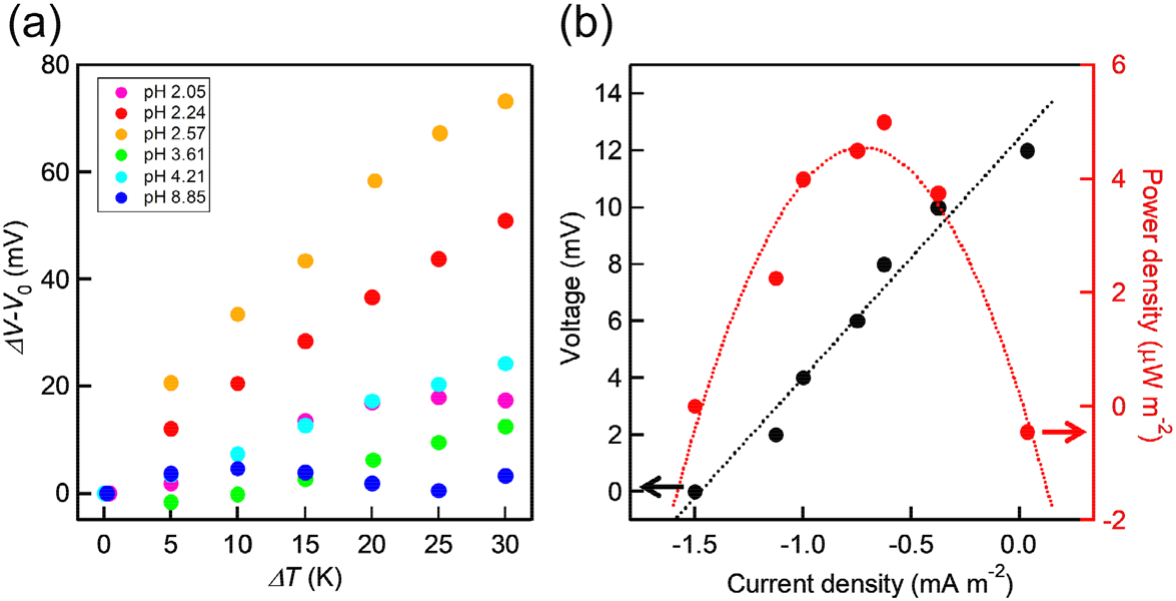
**(a)** pH dependence of *ΔV* − *V*_0_ and *ΔT* of the TEC cell. *V*_0_ is the initial open-circuit potential difference, as shown in Table S2. The initial concentration of pyruvic acid, lactic acid, and sodium sulfate was 20, 20, and 50 mM, respectively. **(b)** Current output and the corresponding power output. The temperature difference was 10 K. (cold side at 291 K, hot side at 301 K). Dotted curves are inserted as an eye guide.

Figure 4a represents the pH-dependence of *ΔV* − *V*_0_. The slope of the lines increases with a decrease in pH values. At low pH conditions, a large number of protons interact with the TiO_2_ surface and pyruvic acid molecules are associated with the protonated TiO_2_ surface, according to the reaction pathway proposed in the DFT study (Fig. 3). In contrast, in the high pH region, the number of adsorbed protons possibly decreases and the H associations, which are illustrated in Fig. 3, hardly occur, resulting in a low *S*_e_ value. Thus, we conclude that the *S*_e_ value relates to the interaction between substrate molecules and the protonated TiO_2_ surface.

The output power was evaluated from the *I*-*V* curve. Figure 4b reveals a relationship between current density (or power density) and the cell potential. *ΔT* between the electrodes was kept to be 10 K. A linear relationship was observed between the cell voltage and the current. The obtained power density was 5 μW m^−2^ in the TEC cell and smaller than the reported values for the other TEC cells^12^. To clarify the reason for the small power density, cell resistance was measured. The resistance of the cell was 0.015 MΩ, which is smaller than that estimated from the *I*-*V* plot (0.40 MΩ). Then, we attribute the small power density to the large resistance resulting from a high barrier for the transformation of redox species in our TEC cell and concentration polarization due to the smaller generation rate of pyruvic acid than that of lactic acid. Therefore, a large power density is probably achievable by optimizing electrolyte and cell design.

To evaluate the long-term stability, we continuously monitored current density and power density for 24 h, as shown in Fig S8. The power density gradually decreased for the first 2 h and then the constant value was maintained. The small power density is due to the slow oxidation reaction compared to that in cathodic side, according to the cyclic voltammogram shown in Figs. S9 and S10. The large power density will be maintained by the enhancement of the oxidation reaction.

The versatility of the redox species was also investigated. Fig. S11 shows the *ΔV* − *V*_0_ and *ΔT* between two electrodes in the TEC cell employing oxalic acid and glycolic acid as the redox couples. The *ΔV* − *V*_0_ increased as the *ΔT* increased. Therefore, this result opens the perspective that various α-keto acids might be utilized as a redox couple for the TiO_2_/Ti mesh electrode.

## Conclusions

In summary, we first demonstrate the TEC conversion using a biocompatible organic redox couple, i.e., pyruvic and lactic acids, and the TiO_2_/Ti mesh electrode with 1.40 mV K^−1^ of a positive *S*_e_ value. The DFT calculation revealed that the positive *S*_e_ value is ascribed to the difference in stability between lactic acid and pyruvic acid, which are adsorbed on the protonated TiO_2_ surface. Furthermore, the TiO_2_ surface acting as a proton donor (or acceptor) plays a critical role in determining the polarity of the generated potential. This result indicates that the interaction between redox species and electrode surface is considerably important in redox-based TEC conversion systems. Our findings suggest that favorable energy conversions will be achievable by designing redox couples and the electrode surface.

## Experimental

### Materials

Lactic acid, pyruvic acid, and glycolic acid were purchased from Tokyo Chemical Industry Co., Ltd. (TCI). Oxalic acid and sodium sulfate, anhydrous were purchased from Kishida Chemical Co., Ltd. Ti meshes (100 mesh) were provided by Manabe Industry Co., Ltd. All chemicals were used without purification.

### Preparation of electrode

TiO_2_/Ti meshes were prepared by two-step hydrothermal reactions that we reported previously^15^. 1 M NaOH solution was transferred to a 50 mL Teflon-lined stainless steel autoclave. Then titanium meshes (2 × 2.5 cm^2^) were placed at Teflon-lined stainless steel autoclave. The hydrothermal treatment was conducted at 220 °C for 12 h. After the reaction, the autoclave was cooled to room temperature and the obtained titanate mesh was immersed in 0.1 M HCl solution for 10 min. Then, titanate mesh rinsed with deionized water. The deionized water was transferred to a 50 mL Teflon-lined stainless steel autoclave and titanate meshes were placed at Teflon-lined stainless steel autoclave. The hydrothermal treatment was conducted at 200 °C for 24 h. After the reaction, the autoclave was cooled to room temperature and the obtained TiO_2_ meshes were rinsed with deionized water.

### Characterization of electrode

X-ray diffraction (XRD) pattern was measured using Rigaku SmartLab diffractometer equipped with a Cu-Kα X-ray source (*λ* = 1.5418 Å).

### Measurement for the open-circuit potential of TEC cells

A typical method of measurement for open-circuit potential is described below. As an electrolyte, lactic acid (20 mM), pyruvic acid (20 mM) were introduced into an H-shaped glass cell (Fig. S12 and sodium sulfate (50 mM) was added as a supporting electrolyte. The temperature of each branch was controlled by using a water bath. The solution was stirred by a magnetic stir bar. Two TiO_2_/Ti mesh electrodes were separately soaked into the cell as shown in Fig. S12. The open-circuit voltage was measured as the potential difference between the two electrodes by Keithley 2401 source meter. The pH value of the solution was adjusted by introducing the aqueous solution of NaOH and/or sulfuric acid. The TiO_2_/Ti mesh electrodes used for the measurement were put into pure water overnight with connected with the chips and wire before use.

### Evaluation of long term stability

The long term stability was evaluated using an H-shaped glass cell. As an electrolyte, lactic acid (20 mM), pyruvic acid (20 mM), and sodium sulfate (50 mM) were introduced into the cell. The temperature of each branch was controlled by using a water bath. The hot side was kept at 306 K and the cold side was kept at 296 K. Two TiO_2_/Ti mesh electrodes were separately soaked into the cell, and the voltage of 8 mV was applied by the VersaSTAT 3 potentiostat (Princeton Applied Research).

### Cyclic voltammetry (CV) measurement

Cyclic voltammetry measurement was conducted using VersaSTAT 3 potentiostat (Princeton Applied Research) and a Teflon-caped glass cell. As an aqueous electrolyte, lactic acid (20 mM), pyruvic acid (20 mM), and sodium sulfate (50 mM) were introduced into the cell. TiO_2_/Ti mesh, Ag/AgCl, and coiled Pt wire were soaked into the glass cell and used as working, reference, and counter electrodes, respectively. The current was recorded under a swept potential at a scan rate of 10 mV s^−1^.

### Computational details

All calculations performed in this study are based on the plane wave DFT method implemented in the Vienna Ab initio Simulation Package (VASP 5.4.4)^28–30^. Perdew − Burke − Ernzerhof parametrization under the generalized gradient approximation was employed as an exchange–correlation functional together with the projector augmented wave method^31^. Spin-polarized calculations were performed throughout the study with a plane wave cutoff energy of 500 eV. The convergence criteria for all calculations were set until the difference in total forces between two ionic steps was less than 0.02 eV/Å, and 10^−5^ eV/atom for the self-consistent field iterations. In this work, to correctly estimate the electronic properties of the Ti atoms, the GGA + U approximation with Hubbard parameter for Ti of 3.0 eV was selected, as it showed to fit the redox properties of TiO_2_.^32^ The optimization of the bulk TiO_2_ anatase phase was performed with 15 × 15 × 6 Monkhorst − Pack k-point mesh for the Brillouin zone integration where all the atoms and the crystal volume were allowed to relax. After the optimization, the calculated lattice parameters of bulk TiO_2_ (*a* = *b* = 3.882 Å, and *c* = 9.663 Å), and the length of the Ti − O apical bond (*d* = 2.008 Å) are in good agreement with the experimental values (*a* = *b* = 3.782 Å, *c* = 9.502 Å, and *d* = 1.979 Å)^33^. Subsequently, the nine layers TiO_2_(001) slab containing a total of 108 atoms was modeled. The TiO_2_(001) surface was selected as it was suggested that the shape of the anatase nanocrystallites is a truncated bipyramid exposing the (101) and (001) surfaces^34^. The slab was optimized with 3 × 3 × 1 Monkhorst − Pack k-point mesh. To avoid dipole − dipole interaction between the sides of the slab, the coordinates of the atoms in the middle three layers of the slab were kept frozen while the coordinates of the atoms in the three top layers for both sides of the slab were fully relaxed. The vacuum was set to span a range of 15 Å to ensure no significant interaction between the slabs, as shown in Fig. S5. To incorporate the effect of the presence of solvent, the self-consistent implicit solvation model incorporated in the VASPsol 2.0 software package was employed^35,36^. The dielectric constant of water was set to 75.70, which corresponds to the experimental dielectric constant at a temperature of 303 K.^37,38^The H atoms binding energy, ${E}_{bind}$, was defined as:

4 $${E}_{bind}={E}_{slab+H}-[{E}_{slab}+{2E}_{H\,\,atom}]$$

where ${E}_{slab+H}$, ${E}_{slab}$, and ${E}_{H\,\,atom}$ are the total energies of the H atoms interacting with the TiO_2_(001) surface, the pristine TiO_2_(001) surface, and the isolated H atom, respectively. The two H atoms interaction leading to the formation of two OHs on the TiO_2_(001) surface is thermodynamically favored with a binding energy of − 5.89 eV, and -5.86 eV when the solvent effect is considered. The pyruvic acid adsorption, ${E}_{ads}$, energy was calculated as follows:

5 $${E}_{ads}={E}_{slab+H+pyruvic}-[{E}_{slab+H}+{E}_{pyruvic}]$$

where ${E}_{slab+H+pyruvic}$, and ${E}_{pyruvic}$ are the total energies of the pyruvic acid molecule interacting with the protonated TiO_2_(001) surface, and the isolated pyruvic acid molecule, respectively. The adsorption energy of the pyruvic acid on the protonated TiO_2_(001) surface is − 1.33 eV and − 0.82 eV if the solvent effect is considered. The relative energy, ${E}_{rel}$, of the intermediates formed was calculated as follows:

6 $${E}_{rel}={E}_{int}-[{E}_{slab+H+pyruvic}]$$

where ${E}_{int}$, corresponds to the total energies of the reaction intermediates after the H atom association occurred. In all cases, a negative value of the binding, adsorption, and relative energies denotes a stable interaction. To assess the trend in stability change of the overall chemical reaction, the intermediate states were calculated without the transition state search.

## Supplementary Information

Supplementary Information.

## References

[1] Chu S, Majumdar A (2012). Opportunities and challenges for a sustainable energy future. Nature.

[2] Taroni PJ, Hoces I, Stingelin N, Heeney M, Bilotti E (2014). Thermoelectric materials: A brief historical survey from metal junctions and inorganic semiconductors to organic polymers. Israel J. Chem..

[3] Bubnova O, Khan ZU, Malti A, Braun S, Fahlman M, Berggren M, Crispin X (2011). Optimization of the thermoelectric figure of merit in the conducting polymer poly(3,4-ethylenedioxythiophene). Nat. Mater..

[4] Toshima N (2017). Recent progress of organic and hybrid thermoelectric materials. Synth. Met..

[5] Quickenden TI, Mua Y (1995). A review of power generation in aqueous thermogalvanic cells. J. Electrochem. Soc..

[6] Dupont MF, MacFarlane DR, Pringle JM (2017). Thermo-electrochemical cells for waste heat harvesting – progress and perspectives. Chem. Commun..

[7] Hu R (2010). Harvesting waste thermal energy using a carbon-nanotube-based thermo-electrochemical cell. Nano Lett..

[8] Abraham TJ, MacFarlane DR, Pringle JM (2011). Seebeck coefficients in ionic liquids—Prospects for thermo-electrochemical cells. Chem. Commun..

[9] Abraham TJ, MacFarlane DR, Pringle JM (2013). High Seebeck coefficient redox ionic liquid electrolytes for thermal energy harvesting. Energy Environ. Sci..

[10] Midgley, D., Reference electrodes for use in the potentiometric determination of chloride. Part II. Quinhydrone electrodes *Analyst* **109**, 445–452 (1984).

[11] Zhou H, Liu P (2018). High Seebeck coefficient electrochemical thermocells for efficient waste heat recovery. ACS Appl. Energy Mater..

[12] Duan J (2018). Aqueous thermogalvanic cells with a high Seebeck coefficient for low-grade heat harvest. Nat. Commun..

[13] Watanabe R, Yamauchi M, Sadakiyo M, Abe R, Takeguchi T (2015). CO_2_-free electric power circulation via direct charge and discharge using the glycolic acid/oxalic acid redox couple. Energy Environ. Sci..

[14] Fukushima T, Kitano S, Hata S, Yamauchi M (2018). Carbon-neutral energy cycles using alcohols. Sci. Technol. Adv. Mater..

[15] Sadakiyo M, Hata S, Cui X, Yamauchi M (2017). Electrochemical production of glycolic acid from oxalic acid using a polymer electrolyte alcohol electrosynthesis cell containing a porous TiO_2_ catalyst. Sci. Rep..

[16] Fukushima T, Higashi M, Kitano S, Sugiyama T, Yamauchi M (2020). Multiscale design for high-performance glycolic acid electro-synthesis cell: Preparation of nanoscale-IrO_2_-applied Ti anode and optimization of cell assembling. Catal. Today.

[17] Sadakiyo M, Hata S, Fukushima T, Juhász G, Yamauchi M (2019). Electrochemical hydrogenation of non-aromatic carboxylic acid derivatives as a sustainable synthesis process: From catalyst design to device construction. Phys. Chem. Chem. Phys..

[18] Fukushima T, Yamauchi M (2019). Electrosynthesis of amino acids from biomass-derivable acids on titanium dioxide. Chem. Commun..

[19] Fukushima T, Yamauchi M (2021). Electrosynthesis of glycine from bio-derivable oxalic acid. J. Appl. Electrochem..

[20] Hu R (2010). Harvesting waste thermal energy using a carbon-nanotube-based thermo-electrochemical cell. Nano Lett..

[21] Quickenden TI, Vernon CF (1986). Thermogalvanic conversion of heat to electricity. Sol. Energy.

[22] Alberty RA (1998). Calculation of standard transformed Gibbs energies and standard transformed enthalpies of biochemical reactants. Arch. Biochem. Biophys..

[23] Ben-Tal N, Honig B, Bagdassarian CK, Ben-Shaul A (2000). Association entropy in adsorption processes. Biophys. J..

[24] Ren P (2012). Biomolecular electrostatics and solvation: A computational perspective. Q. Rev. Biophys..

[25] Zhang J, Zhang H, Wu T, Wang Q, van der Spoel D (2017). Comparison of implicit and explicit solvent models for the calculation of solvation free energy in organic solvents. J. Chem. Theory Comput..

[26] Zhang H, Tan T, van der Spoel D (2015). Generalized Born and explicit solvent models for free energy calculations in organic solvents: Cyclodextrin dimerization. J. Chem. Theory Comput..

[27] Pierce, C. & Smith, R. N. Heats of adsorption. IV. Entropy changes in adsorption. *J. Phys. Colloid Chem.* **54**, 795–803 (1950). 10.1021/j150480a007 15422534

[28] Kresse G, Hafner J (1993). *Ab initio* molecular dynamics for liquid metals. Phys. Rev. B.

[29] Kresse, G. & Furthmüller, J. Efficient iterative schemes for *ab initio* total-energy calculations using a plane-wave basis set. *Phys. Rev. B* **54**, 11169–11186 (1996). 10.1103/physrevb.54.11169 9984901

[30] Kresse G, Furthmüller J (1996). Efficiency of ab-initio total energy calculations for metals and semiconductors using a plane-wave basis set. Comput. Mater. Sci..

[31] Blöchl PE (1994). Projector augmented-wave method. Phys. Rev. B.

[32] Hu Z, Metiu H (2011). Choice of *U* for DFT+*U* calculations for titanium oxides. J. Phys. Chem. C.

[33] Burdett JK, Hughbanks T, Miller GJ, Richardson JW, Smith JV (1987). Structural-electronic relationships in inorganic solids: Powder neutron diffraction studies of the rutile and anatase polymorphs of titanium dioxide at 15 and 295 K. J. Am. Chem. Soc..

[34] Bourikas K, Kordulis C, Lycourghiotis A (2014). Titanium dioxide (anatase and rutile): Surface chemistry, liquid–solid interface chemistry, and scientific synthesis of supported catalysts. Chem. Rev..

[35] Mathew, K., Sundararaman, R., Letchworth-Weaver, K., Arias, T. A. & Hennig, R. G. Implicit solvation model for density-functional study of nanocrystal surfaces and reaction pathways. *J. Chem. Phys.* **140**, 084106 (2014). 10.1063/1.4865107 24588147

[36] Mathew, K., Kolluru, V. S. C., Mula, S., Steinmann, S. N. & Hennig, R. G. Implicit self-consistent electrolyte model in plane-wave density-functional theory. *J. Chem. Phys.* **151**, 234101 (2019). 10.1063/1.5132354 31864239

[37] Moldoveanu, S. C. & David, V. Mobile phases and their properties. in *Essentials in Modern HPLC Separations* 363–447. 10.1016/B978-0-12-385013-3.00007-0 (Elsevier, 2013).

[38] Owen BB, Miller RC, Milner CE, Cogan HL (1961). The dielectric constant of water as a function of temperature and pressure 1,2. J. Phys. Chem..

