# Supplementary material for: Inversely polarized thermo-electrochemical power generation via the reaction of an organic redox couple on a TiO2/Ti mesh electrode
Source: Sci Rep. 2021 Jul 6;11:13929. doi: 10.1038/s41598-021-93269-7 (PMC8260709; doi:10.1038/s41598-021-93269-7)
Supplement: Supplementary file 1 — Supplementary Information. [file 41598_2021_93269_MOESM1_ESM.docx]

**Supplementary Information**

**Inversely polarized thermo-electrochemical power generation via the reaction of an organic redox couple on a TiO_2_/Ti mesh electrode**

Hiroto Eguchi,^1^ Takashi Kobayashi,^2^ Teppei Yamada,^2, 3^* David S. Rivera Rocabado,^4^ Takayoshi Ishimoto,^4, 5^* Miho Yamauchi^1, 6, 7^*

^1^Department of Chemistry, Graduate School of Science, Kyushu University, Motooka 744, Nishi-ku Fukuoka 819-0395, Japan.

^2^Division of Chemistry and Biochemistry, Graduate School of Science, Kyushu University, Motooka 744, Nishi-ku, Fukuoka 819-0395, Japan.

^3^Center for Molecular Systems, Kyushu University, Motooka 744, Nishi-ku, Fukuoka 819-0395, Japan.

^4^Graduate School of Nanobioscience, Yokohama City University, Seto 22-2, Kanazawa-ku Yokohama 236-0027, Japan.

^5^Department of Applied Chemistry, Graduate School of Engineering, Hiroshima University, Kagamiyama 1-4-1, Higashi-Hiroshima, Hiroshima 739-8527, Japan.

^6^International Institute for Carbon-Neutral Energy Research (WPI-I^2^CNER), Kyushu University, Motooka 744, Nishi-ku, Fukuoka 819-0395, Japan6

^7^Advanced Institute for Materials Research (WPI-AIMR), Tohoku University, 2-1-1 Katahira, Aoba-ku, Sendai, 980-8577 Japan


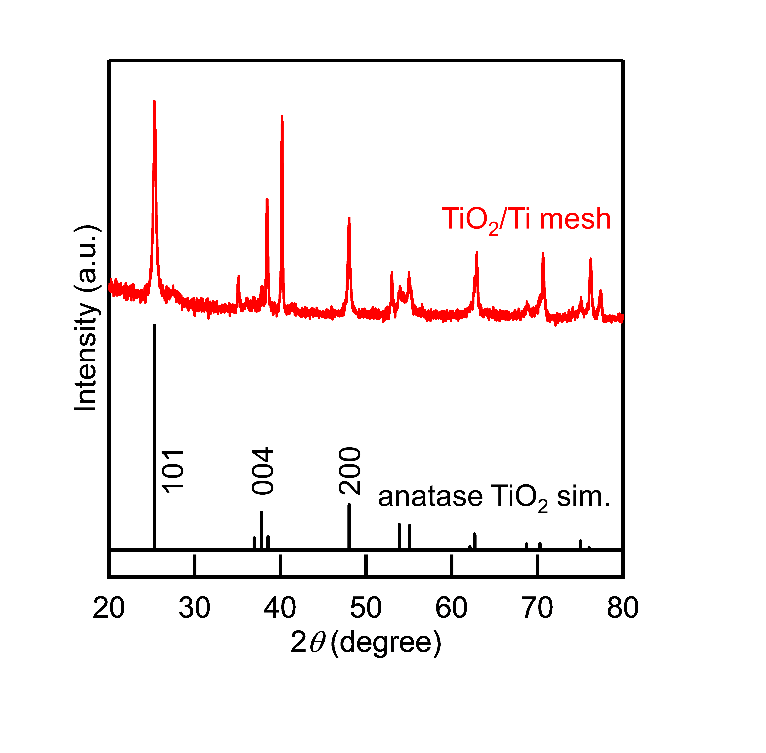


Fig. S1 XRD patterns of a TiO_2_/Ti mesh electrode.


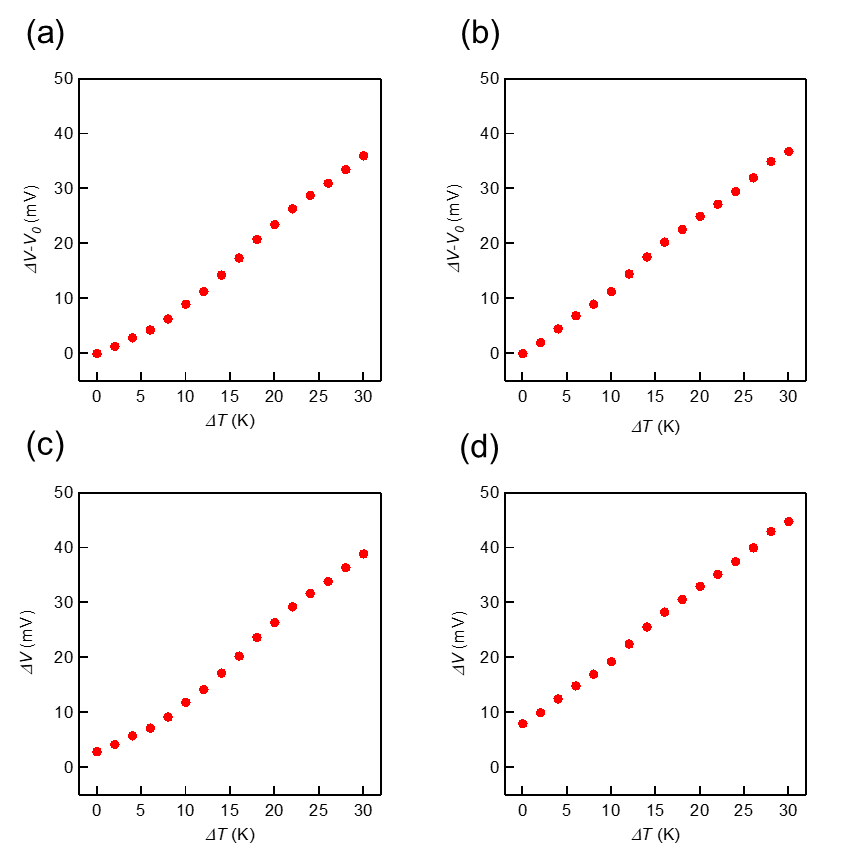
Fig. S2 (a), (b) The difference in open-circuit potential (*ΔV*−*V*_0_) and temperature difference (*ΔT*) of the TEC cell. *V*_0_ is the initial open-circuit potential difference as shown in Table S2. (c), (d) The open-circuit potential (*ΔV*) and temperature difference (*ΔT*) of the TEC cell. The initial concentration of pyruvic acid, lactic acid, and sodium sulfate was 20, 20 and 50 mM, respectively.

Fig. S3 The open-circuit potential (*ΔV*) and temperature difference (*ΔT*) of the TEC cell in Figure 2. The initial concentration of pyruvic acid, lactic acid, and sodium sulfate was 20, 20 and 50 mM, respectively.


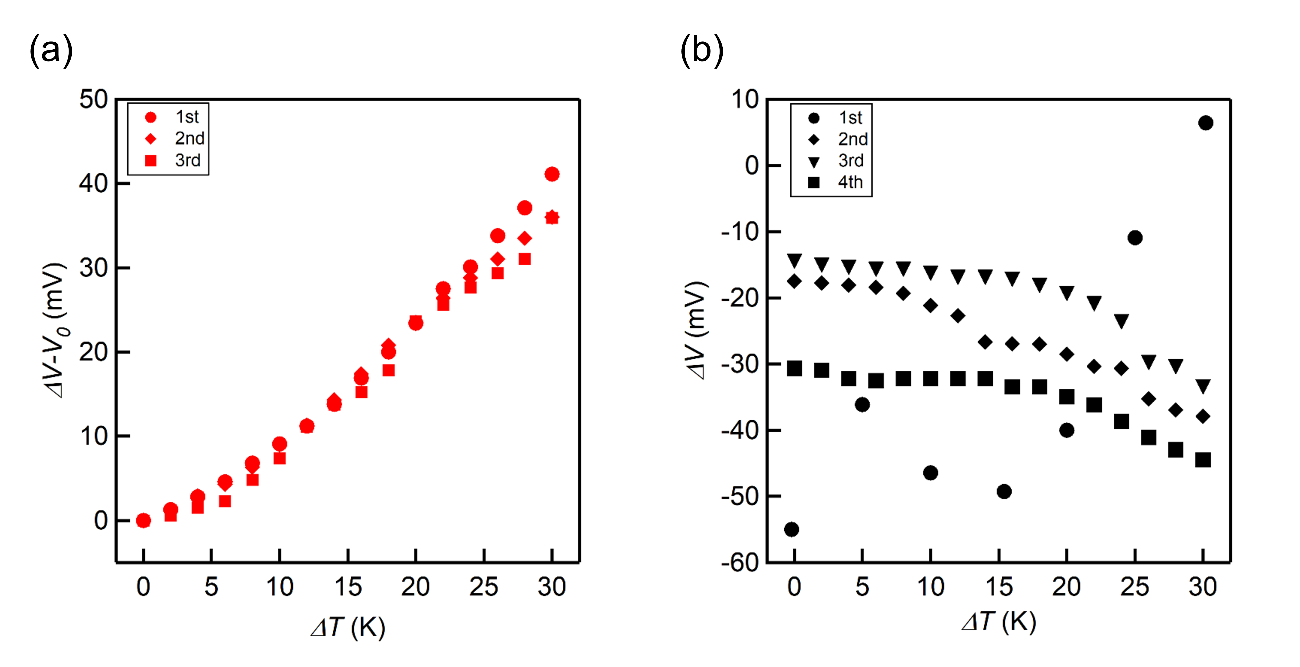


Fig. S4 (a) Difference in the open-circuit potential (*ΔV*−*V*_0_) and temperature difference (*ΔT*) of the TEC cell. *V*_0_ is the initial open-circuit potential differences. The initial concentration of pyruvic acid, lactic acid, and sodium sulfate was 20, 20 and 50 mM, respectively. For the 3rd measurement, newly fabricated cell was used. (b) Difference in the open-circuit potential (*ΔV*) and temperature difference (*ΔT*) of the TEC cell without pyruvic acid and lactic acid using different TiO_2_/Ti mesh electrode. The initial concentration of sodium sulfate was 50 mM. The 2nd, 3rd and 4th measurements were conducted in a newly fabricated cell.


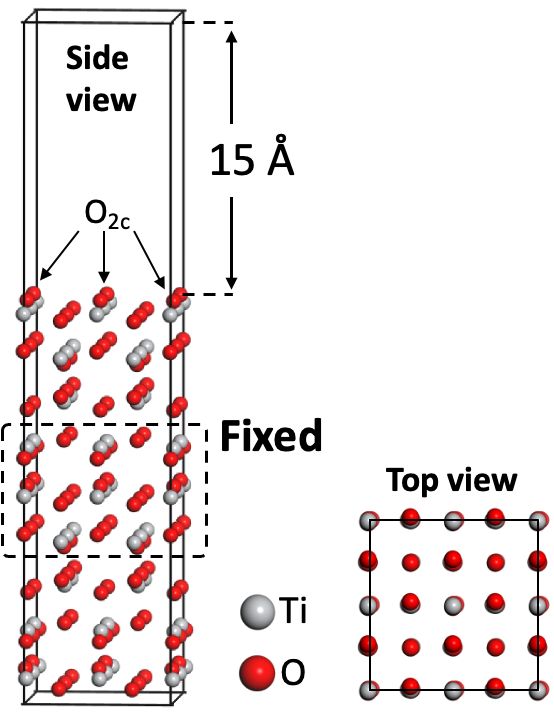


Fig. S5 Side and top views of the nine layered TiO_2_(001) slab.


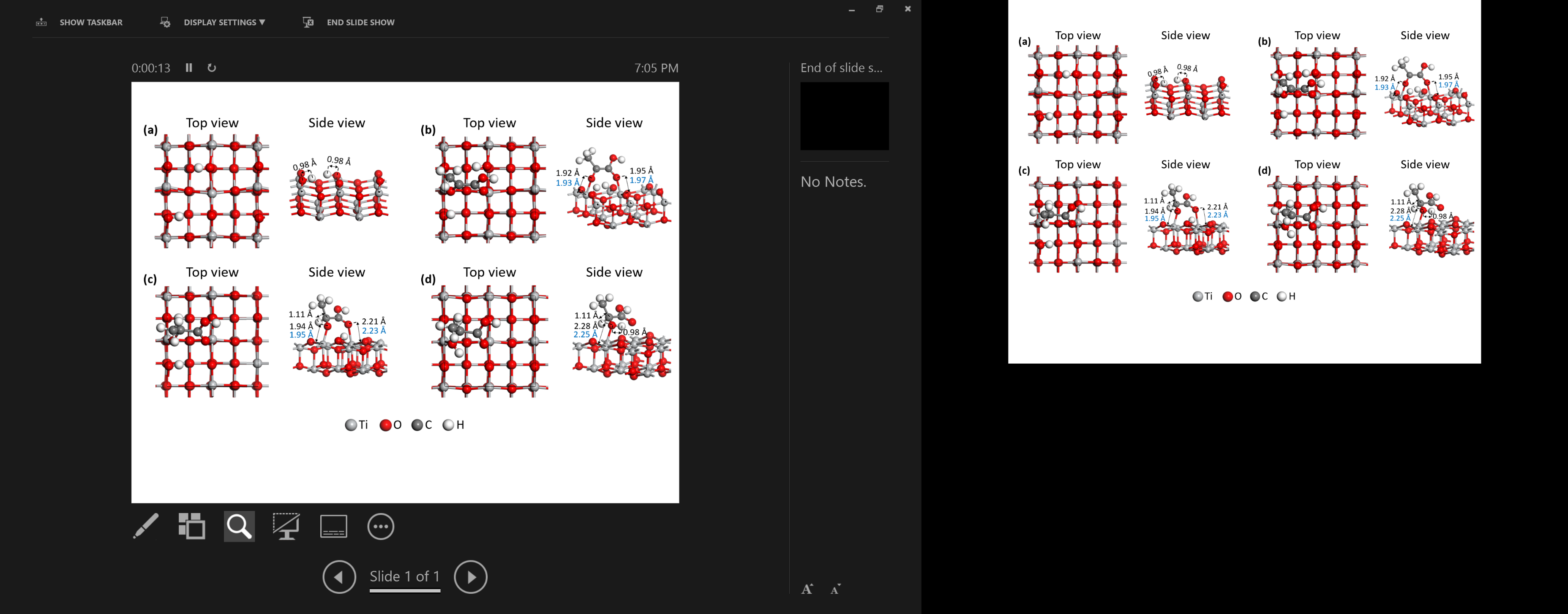
Fig. S6 Top and side views: (a) protonated TiO_2_(001), (b) pyruvic acid adsorption, (c) first H atom association, and (d) second H atom association (lactic acid formation) on the protonated TiO_2_(001). Only surface and subsurface atoms are shown for the side views. The most relevant bonds are shown. The blue values correspond to the change in the interatomic distance because of the solvent effect consideration.


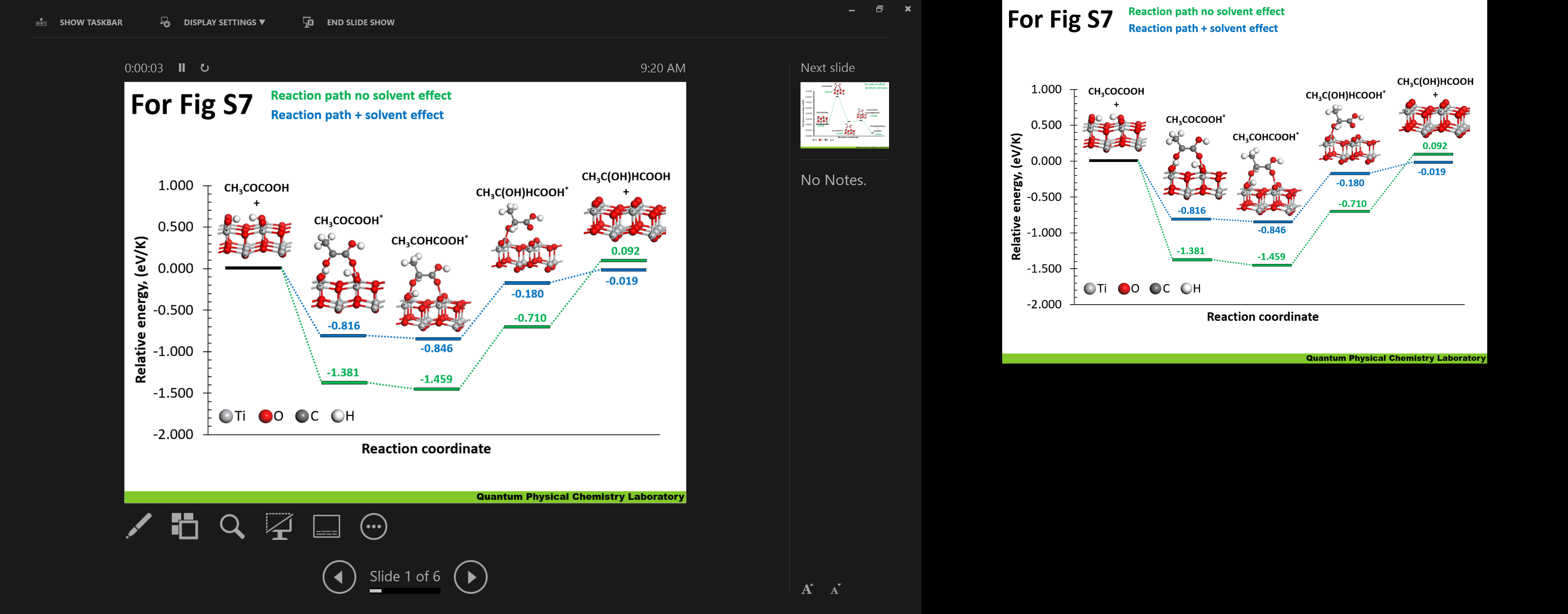


Fig. S7 Reaction pathway of the pyruvic acid reduction to lactic acid on the protonated TiO_2_(001). Blue and green paths indicate the reaction with and without the solvent effect consideration, respectively.


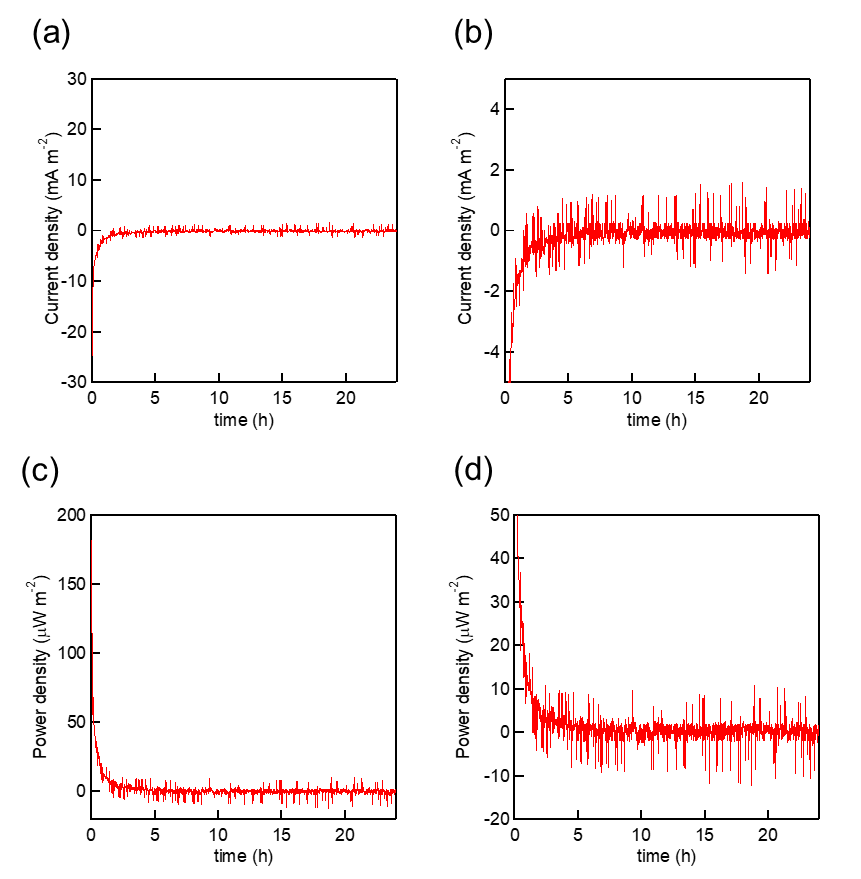


Fig. S8 (a) Time course of the current density of the TEC cell. (b) The enlarged figure of Fig S7 (a). (c) Time course of the power density of the TEC cell. (d) The enlarged figure of Fig S7(c). *ΔT* was kept at 10.0 K and the temperature of the hot side kept to be 306 K. The initial concentration of lactic acid, pyruvic acid, and sodium sulfate was 20, 20, and 50 mM, respectively.

Fig. S9 (a) Cyclic voltammogram at a 293 K. Initial concentrations of pyruvic acid, lactic acid, and sodium sulfate were 20, 20, and 50 mM, respectively. (b) The enlarged figure of cyclic voltammogram.
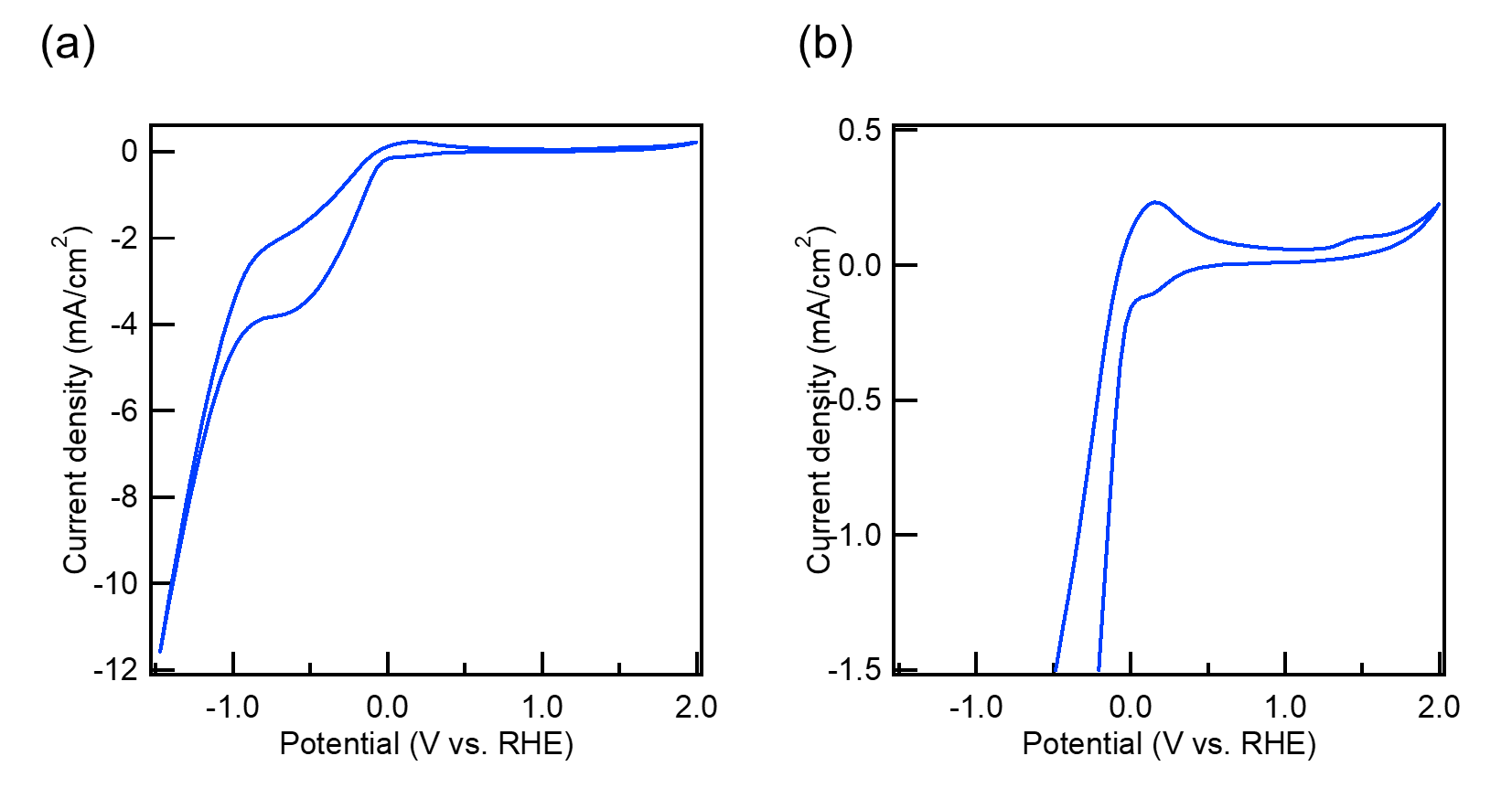

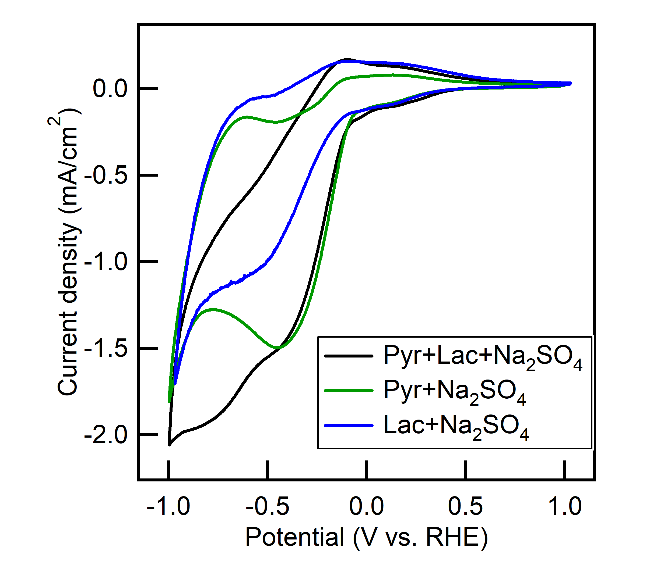
Fig. S10 Cyclic voltammograms in a potential range of -1.0–1.0 V vs. RHE on TiO_2_/Ti mesh electrodes using the solution of 20 mM pyruvic acid, 20 mM lactic acid and 50 mM sodium sulfate (black line), 20 M lactic acid and 50 mM sodium sulfate (blue line) and 20 mM pyruvic acid and 50 mM sodium sulfate (green line).

To clarify the origin of reduction and oxidation current, we conducted cyclic voltammetry in the narrower potential range of –1.0 to 1.0 V vs. RHE with several combinations of substrates and electrolytes as shown in Fig. S10. First, we compare the results obtained in the solutions of pyruvic acid (Pyr) and lactic acid (Lac). The reduction current observed in the range of –0.5 to 0 V vs. RHE seemed to depend on the concentration of pyruvic acid, i.e., current density for Pyr was larger than that for Lac, which clearly suggests that reduction of pyruvic acid occurs. In contrast, the current density in the sweep from –0.6 to 0.5 V vs. RHE depended on the concentration of lactic acid, i.e., current density for Lac was larger than that for Pyr. Thus, we think that this system is electrochemically reversible. However, the oxidation current was smaller than the reduction current for all cases. In the mixed solution of pyruvic acid and lactic acid (Pry+Lac), which includes a higher concentration of substrates than the others, the reduction current continuously increased below –0.5 V vs. RHE. These complex phenomena probably come from the difference in the absorption energy of substrate molecules, i.e., α-keto acid such as pyruvic acid shows high affinity to TiO_2_ surface, which was discussed in our previous report (Phys. Chem. Chem. Phys., 21, 5882-5889 (2019)). In the mixed solution, the reduction of pyruvic acid smoothly proceeds because of its higher affinity and the concentration of pyruvic acid decreases in sweeping to a negative potential. Below –0.5 V vs. RHE, the concentration of lactic acid having weaker interaction becomes higher and oxonium ions are able to access to negatively charged catalyst surface, resulting in hydrogen evolution. Therefore, it is probable that hydrogen evolution easily occurs at higher lactic acid concentrations. Thus, the asymmetry in CV curves is ascribed to two factors, i.e., the difference in affinity of substrate molecules and overlapping with irreversible hydrogen evolution.


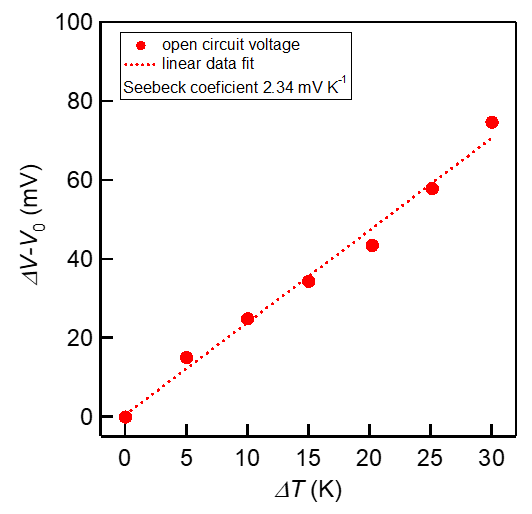
Fig. S11 *ΔV*−*V*_0_ and *ΔT* of the TEC cell. *V*_0_ is the initial open-circuit potential difference, as shown in Table S2. The initial concentration of oxalic acid, glycolic acid, and sodium sulfate was 20, 20, and 50 mM, respectively. The slope of the plot was 2.34 mV K^-1^.


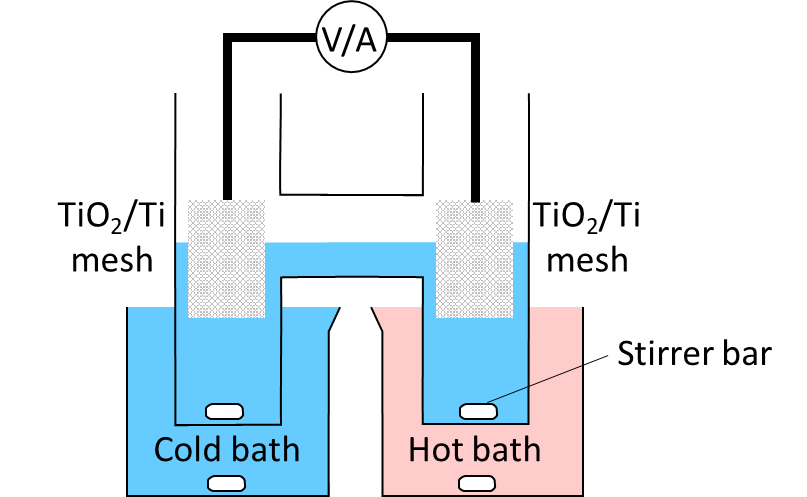


Fig. S12 Schematic illustration of the H-shaped grass cell. The left side of the glass tube was soaked into a cold bath and the right side of the glass tube was soaked into a hot bath.

Table S1 Reported Seebeck coefficient values

| redox couple | Seebeck coefficient *S*_e_ (mV K^-1^) | reference |
| --- | --- | --- |
| ferri/ferrocyanide (Fe(CN)_6_^3−^/Fe(CN)_6_^4−^) | −1.4 | [S1] |
| iodide/triiodide(I^−^/I_3_^−^) | 0.53 | [S2] |
| cobalt(II/III) tris(bipyridyl) (Co^2+/3+^ (bpy)_3_) | 2.19 | [S3] |
| quinone/hydroquinone (quinhydrone) | -0.63 | [S4] |
| lactic acid/pyruvic acid | 1.40 | this work |

Table S2 Initial open-circuit potential (*V*_0_) of a thermo-electrochemical cell.

| Figure number | Solution | Electrode | pH | *V*_0_ (mV) |
| --- | --- | --- | --- | --- |
| Figure 2 | 20 mM lactic acid, 20 mM pyruvic acid and 50 mM sodium sulfate | TiO_2_/Ti mesh | 2.28 | −2.1 |
| Fig. S2 (a) | 20 mM lactic acid, 20 mM pyruvic acid and 50 mM sodium sulfate | TiO_2_/Ti mesh | 2.28 | 2.9 |
| Fig. S2 (b) | 20 mM lactic acid, 20 mM pyruvic acid and 50 mM sodium sulfate | TiO_2_/Ti mesh | 2.28 | 8 |
| Figure 2 | 20 mM lactic acid, 20 mM pyruvic acid and 50 mM sodium sulfate | Pt wire | 2.28 | 8.0 |
| Figure 4(a) | 20 mM lactic acid, 20 mM pyruvic acid and 50 mM sodium sulfate | TiO_2_/Ti mesh | 2.05 | 5.3 |
| Figure 4(a) | 20 mM lactic acid, 20 mM pyruvic acid and 50 mM sodium sulfate | TiO_2_/Ti mesh | 2.24 | 55.9 |
| Figure 4(a) | 20 mM lactic acid, 20 mM pyruvic acid and 50 mM sodium sulfate | TiO_2_/Ti mesh | 2.57 | 96.9 |
| Figure 4(a) | 20 mM lactic acid, 20 mM pyruvic acid and 50 mM sodium sulfate | TiO_2_/Ti mesh | 3.61 | 11.0 |
| Figure 4(a) | 20 mM lactic acid, 20 mM pyruvic acid and 50 mM sodium sulfate | TiO_2_/Ti mesh | 4.21 | −0.5 |
| Figure 4(a) | 20 mM lactic acid, 20 mM pyruvic acid and 50 mM sodium sulfate | TiO_2_/Ti mesh | 8.85 | −21.6 |
| Fig. S11 | 20 mM oxalic acid, 20 mM glycolic acid and 50 mM sodium sulfate | TiO_2_/Ti mesh | 2.05 | −64.8 |

References

1. Hu R., *et al.* Harvesting waste thermal energy using a carbon-nanotube-based thermo-electrochemical cell. *Nano Lett.* **10**, **3**, 838-846 (2010)
2. Abraham, T. J., MacFarlane, D. R. & Pringle, J. M., Seebeck coefficients in ionic liquids –prospects for thermo-electrochemical cells. *Chem. Commun*. **47**, 6260-6262 (2011).
3. Abraham, T. J., MacFarlane, D. R., & Pringle, J. M., High Seebeck coefficient redox ionic liquid electrolytes for thermal energy harvesting. *Energy Environ. Sci.* **6**, 2639 (2013).
4. Midgley, D., Reference electrodes for use in the potentiometric determination of chloride. part II. quinhydrone electrodes *Analyst* **109**, 445-452 (1984).
